# Intraoperative Analysis of Flow Dynamics in Arteriovenous Composite Y Grafts

**DOI:** 10.5935/1678-9741.20160053

**Published:** 2016

**Authors:** Heraldo Guedis Lobo Filho, José Glauco Lobo Filho, Matheus Duarte Pimentel, Bruno Gadelha Bezerra Silva, Camylla Santos de Souza, Marília Leitão Montenegro, Maria Cláudia de Azevedo Leitão, Francisco Vagnaldo Fechine Jamacuru

**Affiliations:** 1 Universidade Federal do Ceará (UFC), Fortaleza, CE, Brazil; Hospital de Messejana Dr. Carlos Alberto Studart Gomes, Fortaleza, CE, Brazil.; 2 Department of Surgery, Universidade Federal do Ceará (UFC), Fortaleza, CE, Brazil.; 3 Universidade Federal do Ceará (UFC), Fortaleza, CE, Brazil.; 4 Christus College, Fortaleza, CE, Brazil.; 5 Center for Research and Development of Medicines of Universidade Federal do Ceará(UFC), Fortaleza, CE, Brazil.

**Keywords:** Coronary Artery Bypass, Off-Pump, Flowmeters, Mammary Arteries, Saphenous Vein

## Abstract

**Objective:**

Composite graft of left internal thoracic artery and great saphenous vein in revascularization of the left coronary system is a technique well described in literature. The aim of this study is to analyze blood flow dynamics in this configuration of composite graft especially in what concerns left internal thoracic artery's adaptability and influence of great saphenous vein segment on left internal thoracic artery's flow.

**Methods:**

Revascularization of left coronary system with composite graft, with left internal thoracic artery revascularizing the anterior interventricular artery and a great saphenous vein segment, anastomosed to the left internal thoracic artery, revascularizing another branch of the left coronary system, was performed in 23 patients. Blood flow was evaluated by transit time flowmetry in all segments of the composite graft (left internal thoracic artery proximal segment, left internal thoracic artery distal segment and great saphenous vein segment). Measures were performed in baseline condition and after dobutamine-induced stress, without and with non-traumatic temporary clamping of the distal segments of the composite graft.

**Results:**

Pharmacological stress resulted in increase of blood flow values in the analyzed segments (*P*<0.05). Non-traumatic temporary clamping of great saphenous vein segment did not result in statistically significant changes in the flow of left internal thoracic artery distal segment, both in baseline condition and under pharmacological stress. Similarly, non-traumatic temporary clamping of left internal thoracic artery distal segment did not result in statistically significant changes in great saphenous vein segment flow.

**Conclusion:**

Composite grafts with left internal thoracic artery and great saphenous vein for revascularization of left coronary system, resulted in blood flow dynamics with physiological adaptability, both at rest and after pharmacological stress, according to demand. Presence of great saphenous vein segment did not alter physiological blood flow dynamics in distal segment of left internal thoracic artery.

**Table:** 

Abbreviations, acronyms & symbols				
**AAM**	**=Ascending aorta manipulation**		**ICU**	**=Intensive care unit**
**AIA**	**=Anterior interventricular artery**		**LCS**	**=Left coronary system**
**AMI**	**=Acute myocardial infarction**		**LITA**	**=Left internal thoracic artery**
**ANOVA**	**=Analysis of variance**		**MAP**	**=Mean arterial pressure**
**CABG**	**=Coronary artery bypass graft**		**MF**	**=Mean flow**
**CFR**	**=Coronary flow reserve**		**PI**	**=Pulsatile index**
**CPB**	**=Cardiopulmonary bypass**		**RITA**	**= Right internal thoracic artery**
**DF**	**=Diastolic fraction**		**TTFM**	**= Transit time flowmetry**
**GSV**	**=Great saphenous vein**			

## INTRODUCTION

The composite Y graft using the left internal thoracic artery (LITA), associated to arterial or great saphenous vein (GSV) segments to revascularize the left or the right coronary system has been used to minimize the risk of cerebrovascular accident resulting from ascending aorta manipulation (AAM)^[1-8]^.

Although some authors recommend the use of only arterial composite grafts^[3,9]^, several researchers have described the safety and effectiveness of LITA and GSV composite grafts^[5-7,10,11]^.

Even though it has been demonstrated that the LITA is capable of providing adequate blood flow for two or more arteries from the left coronary system (LCS)^[10,12]^, there is still concern about the possibility of hypoperfusion^[13]^. There is still concern that the presence of the secondary graft of GSV can reduce blood flow in the LITA distal segment, due to phenomena of competition or steal of flow^[14]^.

Transit time flowmetry (TTFM) is the most frequently used method for the evaluation of graft flow and patency in coronary artery bypass surgery (CABG)^[15-17]^. Few studies have evaluated, in the intraoperative period, blood flow dynamics in arteriovenous composite Y grafts with this technique, leaving an important gap in the study of this graft configuration^[9,18-20]^.

This study aims to evaluate blood flow dynamics in LITA and GSV composite Y grafts, especially in what concerns LITA adaptability and the influence of GSV segment on LITA blood flow.

## METHODS

### Study Approval

The study was approved by the Research Ethics Committee (Federal University of Ceará/PROPESQ) under the legal advice number 622589. All patients signed an Informed Consent Form.

### Patients Profile

From July 2013 to June 2015, 23 patients were enrolled in this prospective study. Demographic data and preoperative variables are described in Table 1. Eligibility criteria were patients undergoing elective CABG without associated procedures. Patients undergoing off-pump elective CABG with arteriovenous composite Y- grafts revascularizing anterior interventricular artery (AIA) and another branch of LCS were included. Exclusion criteria were patients diagnosed with diffuse coronary artery disease, patients who underwent associated procedures. Arteriovenous composite Y graft were used mainly to minimize the AAM in the elderly patients, and in those with any evidence of atheromatous disease of this vessel, as well as in patients with comorbidities associated with this entity, such as diabetes, peripheral arterial occlusive disease and carotid artery disease.

**Table 1:** Demographic data and preoperative clinical characteristics of the 23 patients.

Characteristic	Patient (s)
Mean age (years)	64.56±10.21
Age > 75 years	5 (21.73%)
Female	2 (8.69%)
Male	21 (91.3%)
Previous AMI	11 (47.82%)
Functional Class III/IV (NYHA)	3 (13.04%)
BMI	28.14±3.45
Smoking history	12 (52.17%)
COPD	3 (13.04%)
Diabetes	12 (52.17%)
Dyslipidemia	12 (52.17%)
Hypertension	12 (52.17%)
Lesion of left main coronary artery	5 (21.73%)
Two vessel disease	7 (30.43%)
Three vessel disease	11(47.82%)
LVEF (mean)	61.46±10.68
LVEF ≤ 35%	1 (4.34%)
Left ventricular mass index (g/m^2^)	101.46±18.71
Left ventricular mass	185.60±46.45
Cerebrovascular disease	1 (4.34%)
Carotid disease (lesion ≥ 60%)	4 (17.39%)
EuroSCORE	1.03±0.52
STS-SCORE	0.90±0.50
Atrial fibrillation	1 (4.34%)
Creatinine	1.04±0.21

Data described as mean ± standard deviation AMI=acute myocardial infarction; BMI=body mass index; COPD=chronic obstructive pulmonary disease; EuroSCORE=European System for Cardiac Operative Risk Evaluation; LVEF=left ventricular ejection fraction; NYHA=New York Heart Association; STS-score=The Society of Thoracic Surgeons’ Risk Score

### Surgical Technique

All patients underwent off-pump CABG through a median sternotomy. The LITA was harvested in a non-skeletonized way, then bathed in papaverine solution. GSV was harvested using interrupted incisions with skin bridges, atraumatically, and with minimal dilatation. Segments without valves were selected for the construction of the composite graft.

Heparin was administered in the dose of 2 mg/kg. The interruption of blood flow to the anastomosis region was accomplished by snaring, with a 5-0 polypropylene wire and tourniquets, proximally and distally to the sites of the anastomosis. Aiming to protect these vessels, a small silicon tube was interposed between the tourniquets and the coronary artery.

**Fig. 1:**
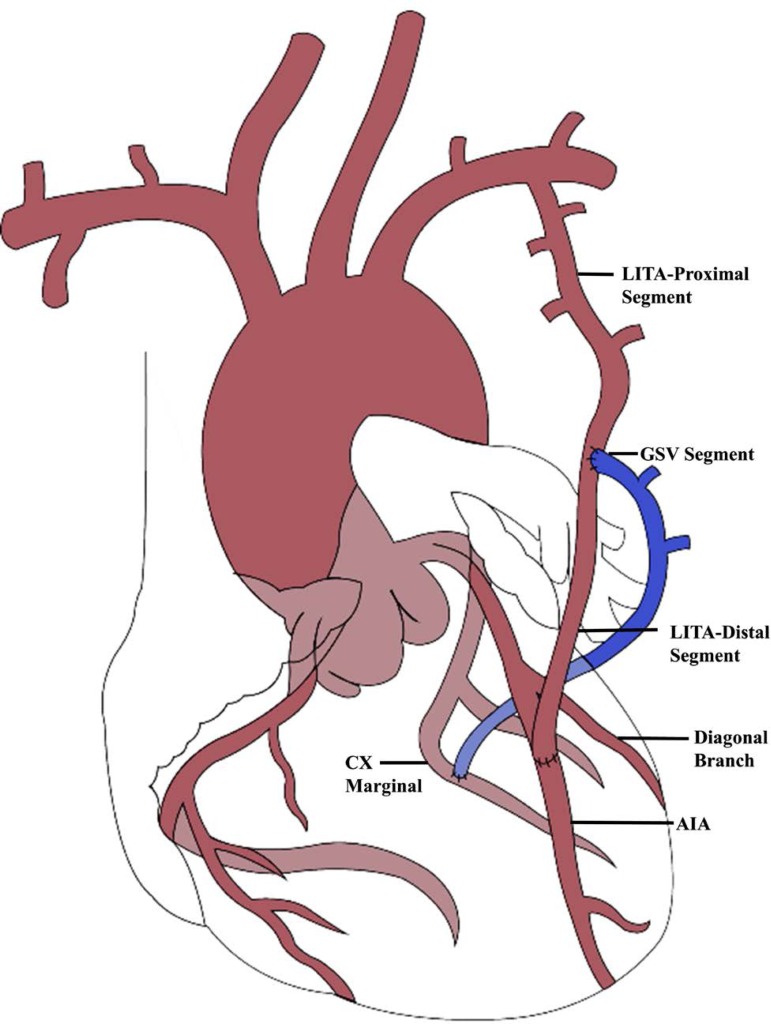
Schematic illustration of the surgical procedure performed in all patients. AIA=anterior interventricular artery; Cx Marginal=marginal branch of circumflex artery; GSV=great saphenous vein; LITA=left internal thoracic artery

A tissue stabilizer was used to perform the coronary anastomosis. Initially, the GSV segment was anastomosed to a diagonal, diagonalis or marginal arterial branch. After that, LITA was anastomosed to the AIA. Finally, the proximal portion of the GSV segment was anastomosed terminal-laterally to the LITA, in a Y configuration. After the flowmetric measurements, protamine was administered to reversion of the heparin (Figure 1, Table 2).

**Table 2:** Artery of left coronary system revascularized by LITA and GSV composite graft.

Graft and coronary artery revascularized	Number of patients	%
LITA - AIA	23	100
GSV - DG	7	30.43
GSV - DGLS	3	13.04
GSV - MGCX	13	56.52

AIA=anterior interventricular artery; DG=diagonal branch of anterior interventricular artery; DGLS=diagonalis branch of left coronary artery; GSV=great saphenous vein; LITA=left internal thoracic artery; MGCX=marginal branch of circumflex artery

### TTFM Measurements

The technique for use of TTFM was well described by D'Ancona et al.^[15]^. After performing all anastomoses, and before the administration of protamine, the analysis of flow in the grafts was performed using a Butterfly flowmeter (Medi-Stim, Oslo, Norway).

To obtain the flowmetric parameters, a probe with appropriate size was placed sequentially:

1. Around the LITA proximal segment;
2. Around the LITA distal segment to collect the data without and with non-traumatic temporary clamping of the GSV segment;
3. Around the GSV segment, to collect the data without and with non-traumatic temporary clamping of the LITA distal segment.

All these measurements were performed initially at baseline condition and repeated after dobutamine infusion (6 µg/kg/min), administered during five minutes, to obtain the values of mean flow (MF), pulsatile index (PI) and diastolic fraction (DF).

Coronary flow reserve (CFR) was calculated by the ratio between MF in stress divided by mean flow in baseline condition, in each segment. During the measurements, mean arterial pressure (MAP) was maintained between 65 and 75 mmHg. All patients enrolled in the study had presented PI ≤ 5 and DF ≥ 60%, which are considered as parameters of patency of the grafts^[15,16]^.

### Statistical Analysis

Flowmetric parameters referring to each segment of the composite graft were initially analyzed by Kolmogorov-Smirnov test to verify the normality of distribution. Mean and standard deviation were calculated and applied for statistical analysis. Comparisons between the flowmetric parameters, considering baseline situation and after pharmacological stress, in the LITA proximal segment, were performed using t test to paired data.

The effects of pharmacological stress (factor 1), clamping of distal LITA and GSV (factor 2) on the MF measured at the distal LITA and at the GSV segment, and the interaction between these two factors, were analyzed by using analysis of variance (ANOVA) for two factors repeated measures associated with *post-hoc* Bonferroni's test.

In all analyses, level of significance was established in 0.05 (5%), being considered as statistically significant a *P* value lesser than 0.05. The software GraphPad Prism^®^ version 5.00 for Windows^®^ (GraphPad Software, San Diego, California, USA, 2007) was used to accomplish statistical procedures.

## RESULTS

**Fig. 2:**
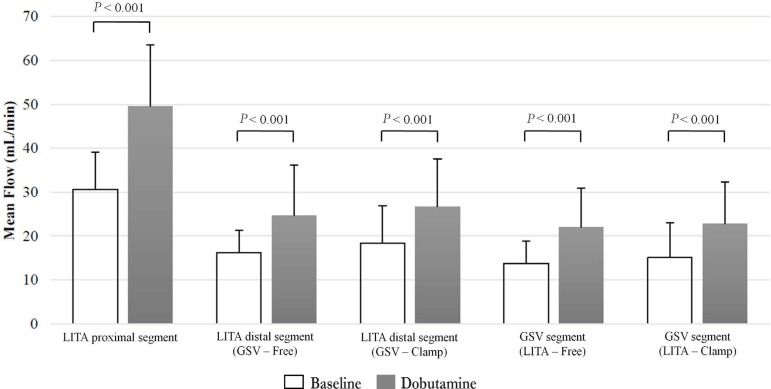
Pharmacological stress influence on blood flow parameter in arteriovenous composite Y graft. GSV=great saphenous vein; GSV-LITA Clamp=GSV segment with clamping of LITA; LITA=left internal thoracic artery; LITA-GSV Clamp=LITA distal segment with clamping of GSV; GSV-LITA Free=GSV segment without clamping of LITA; LITA-GSV Free=LITA distal segment without clamping of GSV

The values of MF for each segment of composite graft, in baseline condition and after the administration of dobutamine, are shown in Figure 2. Without clamping of the distal segments of the composite graft, in baseline condition, LITA proximal segment, LITA distal segment and GSV segment had mean blood flow of 30.65±8.41, 16.22±5.16 and 13.78±5.11 mL/min, respectively. After the administration of dobutamine, flow values changed to 49.57±14.02, 24.70±11.42 and 22.04±8.93 mL/min, respectively. This increase, from baseline condition to stress, was statistically significant (*P*<0.01) for all the evaluated segments of the composite graft.

Clamping of the GSV segment did not result in a statistically significant alteration in mean blood flow values of the LITA distal segment in baseline condition (*P*=0.0633), nor under pharmacological stress (*P*=0.2344) (Table 3).

**Table 3:** Influence of non-traumatic temporary clamping of GSV segment on mean flow values of LITA distal segment.

	Without GSV Clamping	With GSV Clamping	*P*
LITA distal segment baseline flow (mL/min)	16.22±5.16	18.39±8.47	0.0633
LITA distal segment dobutamine flow (mL/min)	24.70±11.42	26.70±10.84	0.2344

Data described as mean ± standard deviation. *P*<0.05 was considered statistically significant GSV=great saphenous vein; LITA=left internal thoracic artery

Likewise, clamping of the LITA distal segment did not result in statistically significant alteration in mean blood flow values of the GSV segment, in baseline condition (*P*=0.2955), or under pharmacological stress (*P*=0.5103) (Table 4).

**Table 4:** Influence of non-traumatic temporary clamping of LITA distal segment on mean flow values of GSV segment.

	Without LITA clamping	With LITA clamping	*P*
GSV segment baseline flow (mL/min)	13.78±5.11	15.04±7.99	0.2955
GSV segment dobutamine flow (mL/min)	22.04±8.93	22.83±9.49	0.5103

Data described as mean ± standard deviation. *P*<0.05 was considered statistically significant GSV=great saphenous vein; LITA=left internal thoracic artery

In the LITA proximal segment, CFR was 1.69±0.50. In the LITA distal segment, CFR was 1.54±0.57 without GSV clamping, and 1.55±0.60, with clamping of this venous segment, without statistical difference (*P*=0.9393). Likewise, in the GSV segment, CRF was 1.66±0.65, without clamping of the LITA distal segment, and 1.61±0.57, with clamping of this arterial branch, also without statistical difference (*P*=0.6613) (Table 5).

**Table 5:** Coronary flow reserve in LITA proximal segment and in distal branches of composite graft.

	LITA proximal segment	LITA distal segment		*P* value	GSV segment		*P* value
		Without GSV clamping	With GSV clamping		Without LITA clamping	With LITA clamping	
CFR	1.69±0.50	1.54±0.57	1.55±0.60	0.9393	1.66±0.65	1.61±0.57	0.6613

Data described as mean ± standard deviation. *P*<0.05 was considered statistically significant CFR=coronary flow reserve; GSV=great saphenous vein; LITA=left internal thoracic artery

All patients had an uncomplicated postoperative recovery. There was no occurrence of acute myocardial infarct (AMI), need for an intra-aortic balloon, cerebrovascular accident, acute renal failure, mediastinitis, osteomyelitis or sepsis. Mean length of intensive care unit (ICU) stay was 2.39±0.58 days, while mean length of hospital stay was 8.13±2.8 days. Patients are currently undergoing outpatient follow-up, without clinical evidences of ischemia.

## DISCUSSION

In the 1980s, Mills and Everson published a study demonstrating the use of LITA and GSV composite graft to revascularize two coronary arteries, in order to avoid AAM in patients with atherosclerotic disease of this vessel^[1]^. Such technique sought to reduce the risks of neurologic complications. In the following decades, several other authors have been presenting their results with similar approaches with good clinical and angiographic outcomes^[3-7,10,11]^.

Several authors have reported series of patients undergoing off-pump CABG without AAM using composite grafts, demonstrating its viability and reproducibility^[4-7,10,11]^.

In this study, flowmetric analysis noted that pharmacological stress resulted in a statistically significant increase of blood flow values in all analyzed segments (*P*<0.05). It was also noted that non-traumatic temporary clamping of the GSV segment did not cause a statistically significant change in the blood flow of the LITA's distal segment, neither at baseline nor under pharmacological stress. Likewise, non-traumatic temporary clamping of the LITA distal segment did not cause a statistically significant change in the blood flow of the GSV segment, in neither baseline condition and under pharmacological stress.

Despite studies demonstrating that the LITA is capable of providing adequate blood flow for two or more LCS arteries, both in baseline situation and in stress^[9,10,12]^, other authors report that composite Y-grafts might present lower CFR than independent grafts^[13]^. CFR, defined as blood flow under stress divided by blood flow in baseline condition, is an effective parameter to evaluate if the graft is capable of providing adequate blood flow in a situation of higher demand. Previous studies show that the LITA in composite grafts increases its diameter^[12,21]^ and flow supply^[9,10,22]^, which means that it presents a morphological and functional adaptation, in addition to possessing a CFR appropriate to supply the myocardium, with values similar to the ones of single grafts^[9,10,21]^. Our study noted that, even in early situations, such as just after the confection of anastomosis, there is a statistically significant increase in blood flow of the LITA proximal segment and of other branches of the composite graft after pharmacological stress. It was also demonstrated by our study that CFR in LITA distal segment has not been modified by the clamping of GSV segment and vice versa. There were no records of clinical and/or laboratorial changes suggesting hypoperfusion.

Gaudino et al.^[9]^, in 2003, investigated, during intraoperative period, the capability of the LITA composite graft, with a right internal thoracic artery (RITA) segment as secondary conduit, to provide appropriate blood flow to the AIA and to a marginal branch of the left circumflex artery, in 21 patients undergoing CABG, ten of which were off-pump. The study of flow in the grafts was performed through TTFM in basal conditions and after pharmacological stress using dobutamine. They observed that blood flow in the interior of graft increased significantly under stress in both the LITA proximal segment, as in the LITA and RITA distal segments. In this study, the influence of the secondary branch of the composite graft in the flow of the LITA was not evaluated^[9]^.

Lobo Filho et al.^[10]^, in 2006, compared, through LITA Dopplerfluxometry performed at least two months after CABG, the blood flow in two groups of patients undergoing this surgical procedure (group A - simple graft, and group B - composite graft). The LITA, as part of an arteriovenous composite Y graft, revascularizing the AIA and another LCS branch, presented a higher mean blood flow when compared to it as a single graft, and also an increase of blood flow from baseline situation to dobutamine-induced stress. There was no significant difference between CFR of both groups (CFR was 1.6 to single graft and 1.8 to composite graft)^[10]^.

In contrast to these findings, Sakaguchi et al.^[13]^, in 2002, whose study evaluated blood flow from regional myocardium through PET scans, observed that, two weeks after the surgical procedure, LITA, when used as a segment of the composite graft, had lower CFR than the one of independent graft. According to data published by Markwirth et al.^[21]^, in 2001, this can be attributed to early flow evaluation, and that the phenomenon of LITA flow adaptability may not occur until the sixth month.

Blood flow into the graft can be also influenced by the utilization of cardiopulmonary bypass (CPB). Balacumaraswami et al.^[19]^, in 2008, in a study involving patients undergoing CABG with different configurations of grafts, noted that patients with on-pump grafts had significantly higher mean blood flow values, as well as lower MAP levels when compared to those whose CABG was performed off-pump. They observed that, for a determinate MAP level, flow into the graft was higher in patients CPB. According to Balacumaraswami et al.^[19]^, this is probably due to the occurrence of a more pronounced vasodilatation after the use of CPB, which can be explained by the release of inflammatory mediators, and by the occurrence of reactive hyperemia in the coronary vascular bed after aortic unclamping.

Żelazny et al.^[20]^, in 2012, published the results of flow analyses by TTFM in patients undergoing off-pump CABG with different configurations of grafts before and after dobutamine-induced pharmacological stress. At baseline, blood flow measured in the ITA proximal portion was higher in the configuration of composite grafts when compared to those found in simple grafts. After dobutamine infusion, all configurations had a significant flow increase, with mean CFR values varying between 1.4 and 2.01^[20]^.

Still in relation to the technique of construction of composite grafts, there is also some concern about the possibility of occurrence of competitive flow and flow steal phenomena, caused by the presence of significant flow in the native coronary and by the GSV segment, which could compromise the results of this approach^[14]^. In our study, blood flow values in the LITA distal segment, at baseline and under pharmacological stress, as well as CFR, were similar when the GSV segment was clamped or not. Likewise, blood flow in the GSV segment, at baseline and under pharmacological stress, as well as CFR, were similar when the LITA distal segment was clamped or not.

Endorsing the above described results, Speziale et al.^[18]^ published, in 2000, a study involving 76 patients undergoing on-pump CABG using arterial composite grafts to revascularize the AIA and another artery of the left or right coronary system. Secondary conduits were radial artery, inferior epigastric artery, right gastroepiploic artery or RITA. Blood flow in composite graft was evaluated by TTFM. When the distal ITA was temporary occluded, blood flow in the other arterial branch did not increase and the same happened to the distal ITA when the other conduit was occluded. As Speziale et al.^[18]^ did not evaluate flow under pharmacological stress, no data could be concluded about CFR.

LITA is universally recognized as the gold standard graft in CABG surgery due to its high patency index, improvement of long term survival, lower necessity of reoperations and lower incidence of myocardial infarction and angina^[23]^. The choice of the secondary conduit to this arterial graft is an important factor for the success of the composite grafting approach. Although some authors are favorable to the exclusive use of arterial grafts^[3,9,18]^, GSV is being widely used by our group^[4,10]^ and others^[5-7,11]^. Some authors compared the LITA and GSV composite graft to others with the LITA with arterial conduits, observing that in both situations early patency rates and within one year of follow-up, as well as clinical results, were similar^[5,11]^.

Despite lower GSV patency in the long term, we believe that it can provide better results when used as secondary conduit in LITA composite grafts, due to the following factors: grafts using small GSV segments without valves, which are predisposed sites for the development of atherosclerotic disease and which can provide a state of hemodynamic stasis and thrombosis^[24]^; GSV anastomosed to the LITA might present less circulatory stress and flow turbulence than when compared to GSV directly anastomosed to the aorta, possibly minimizing the development of venous graft disease^[25]^; in addition to this, it is possible that part of the endothelium protective factors, produced by LITA, may act in benefit of GSV segments^[24,25]^.

### Limitations

This study presents some limitations. Initially, it has a relatively small sample and the patients had a short follow-up. Furthermore, angiographic evaluation of the patients in the follow-up was not performed.

## CONCLUSION

According to the flowmetric analysis, the composite graft with LITA and GSV for revascularization of the AIA and another branch of the LCS respectively, results in blood flow dynamics with physiological adaptability in all areas according to demand, both at rest and after pharmacological stress. The presence of the GSV segment in the disposition of the composite graft in question did not alter the physiological dynamics of blood flow in the distal segment of the LITA.

**Table:** 

Authors’ roles & responsibilities	
HGLF	Analysis and/or data interpretation; conception and design study; manuscript redaction or critical review of its content; realization of operations and/or trials; final manuscript approval
JGLF	Analysis and/or data interpretation; manuscript redaction or critical review of its content; realization of operations and/or trials; final manuscript approval
MDP	Analysis and/or data interpretation; manuscript redaction or critical review of its content; final manuscript approval
BGBS	Analysis and/or data interpretation; manuscript redaction or critical review of its content; final manuscript approval
CSS	Analysis and/or data interpretation; final manuscript approval
MLM	Analysis and/or data interpretation; manuscript redaction or critical review of its content; final manuscript approval
MCAL	Analysis and/or data interpretation; final manuscript approval
FVFJ	Analysis and/or data interpretation; manuscript redaction or critical review of its content; statistical analysis; final manuscript approval
